# Electroacupuncture-augmented breaststroke vs. breaststroke alone for mild AIS: superior correction in a retrospective cohort

**DOI:** 10.3389/fped.2026.1720305

**Published:** 2026-03-03

**Authors:** Xiao Ma, Yuanyuan Zhang, Shilin Lian, Zhuyun Cai, Weihong Li, Tianwen Ye, Rui Gao

**Affiliations:** 1 Department of Orthopedic Surgery, Changzheng Hospital, Naval Medical University, Shanghai, China; 2 Outpatient Department, Changzheng Hospital, Naval Medical University, Shanghai, China; 3 Department of Traditional Chinese Medicine, Changhai Hospital, Naval Medical University, Shanghai, China

**Keywords:** adolescent idiopathic scoliosis, electroacupuncture, swimming, conservative treatment, traditional Chinese medicine

## Abstract

**Background:**

In China, acupuncture is commonly used for adolescent idiopathic scoliosis (AIS), but it requires combination with other therapies. In clinical practice, orthopedic surgeons often recommend swimming for mild AIS patients with Cobb angle <25°. However, owing to the paucity of high-quality evidence and standardized protocols, the efficacy of swimming for AIS remains controversial. The objective of this study was to evaluate the efficacy and safety of breaststroke combined with electroacupuncture vs. breaststroke alone in skeletally immature patients with mild AIS.

**Methods:**

This was a single-center retrospective cohort study. One hundred seventeen mild AIS patients with Risser ≤3 treated from January 2020 to March 2024 were divided into two groups according to different treatments. During the 1-year treatment period, the breaststroke combined with electroacupuncture group (BE-group) received 3 months of electroacupuncture and 12 months of supervised 800-meter breaststroke training, whereas the breaststroke group (B-group) received 12 months of breaststroke training alone. Radiographic parameters, quality of life, and the Walter Reed Visual Assessment Scale (WRVAS) score were evaluated in both cohorts at baseline, 3-month, and 12-month follow-up intervals.

**Results:**

The study included 56 patients in the BE-group and 61 in the B-group. At last follow-up, the BE-group demonstrated superior deformity correction to the B-group in Cobb angle (*p* = 0.003) and WRVAS (*p* < 0.001). Intragroup analysis revealed that, after 12 months of treatment, the BE-group presented significant improvements in Cobb angle (*p* < 0.001), SRS-22 self-image (*p* = 0.003), mental health (*p* = 0.014), WRVAS (*p* < 0.001), as well as a significant reduction in thoracic kyphosis (*p* = 0.014). The B-group demonstrated no significant deformity correction, but maintained curve stability. Both groups showed significant improvements in the SRS-22 pain (*p* < 0.001, *p* = 0.005). For thoracic-dominant AIS, the treatment outcomes showed no significant intergroup difference (*p* = 0.112). However, for thoracolumbar/lumbar-dominant AIS, the BE-group demonstrated superior corrective efficacy (*p* < 0.001).

**Conclusions:**

This study demonstrated that both breaststroke monotherapy and the combined therapy had protective effects on skeletally immature patients with mild AIS. Combining breaststroke with electroacupuncture achieved deformity correction, whereas breaststroke alone merely halted curve progression. Furthermore, the combined therapy was particularly beneficial for thoracolumbar/lumbar-dominant curves.

## Introduction

Scoliosis is a three-dimensional spinal deformity with Cobb angle ≥10° in the coronal plane (1). AIS is the most common type, accounting for 80% of all adolescent patients with scoliosis (2). According to the Scoliosis Research Society (SRS), operative treatment is recommended for patients with a curve greater than 45°, and a brace is used to treat AIS patients with Cobb angle of 25°–40° (3). However, AIS patients who are skeletally immature with curves under 25° present a significant management challenge due to their high risk of curve progression. For these patients, there is no standardized treatment protocol, and clinicians may suggest different treatments, such as observation, exercise, traditional Chinese medicine (TCM), and sports activity.

Paraspinal muscle imbalance plays a significant role in the pathogenesis of scoliosis (4). Our findings also revealed a significant imbalance in muscle volume and fatty infiltration in the deep paravertebral muscles of AIS patients (5). In recent years, a series of studies have explored the effectiveness of acupuncture in treating scoliosis. Acupuncture is defined as the insertion of fine needles through the skin into specific body sites, known as acupuncture points, on meridians to modulate the body's energy flow, called “qi”, and improve body physiology (6). It is believed that tonifying the areas of weak muscles and sedating the areas of overactive muscles through acupuncture stimulation can have relaxing and balancing effects (7). Current evidence suggests that acupuncture has therapeutic effects on scoliosis, but optimal outcomes typically require its combination with other treatments (8). In Hong Kong, other conservative options like spinal manipulation and exercise are widely used to reduce curve progression (9, 10). Electroacupuncture is a modification of acupuncture in which needles with attached electrodes are inserted to deliver a pulsed electrical current (11). Studies indicate that electroacupuncture has superior therapeutic efficacy compared with traditional acupuncture (12). However, current research on electroacupuncture for the treatment of scoliosis is limited.

Sports activity, such as swimming, has a protective role against AIS progression (13). The evidence indicates that the water environment can relieve pressure on the spine and relax muscles (14). Swimming is an attractive exercise method for young people and is helpful in correcting posture defects, alleviating pain, enhancing lung growth, and improving mental health (15–17). Breaststroke represents a symmetrical exercise modality and promotes balanced development of the bilateral muscles (18). The International Society on Scoliosis Orthopedic and Rehabilitation Treatment (SOSORT) guidelines also recommend sports practice as an adjunct to other treatments (2).

This article introduces a new strategy involving electroacupuncture and breaststroke. The aim of this study was to assess the effectiveness and safety of this combinatorial approach in improving the clinical outcomes of mild AIS patients.

## Methods and materials

### Study design

This was a single-center retrospective cohort study. All procedures were performed in accordance with the Declaration of Helsinki and local regulations. The approval for this study was granted by the Ethics Committee of Shanghai Chengzheng Hospital.

### Patient cohort

Of the 834 eligible AIS patients admitted to Shanghai Changzheng Hospital from January 2020 to March 2024, 117 with mild AIS were included in the final study cohort. Patients were divided into two groups according to different interventions. The combined therapy group (BE-group) received 3 months of electroacupuncture and 12 months of supervised breaststroke training, whereas the breaststroke group (B-group) received 12 months of breaststroke training alone. The inclusion criteria were as follows: AIS, aged 10–17 years, Cobb angle of 10°–24°, Risser ≤3, no other treatment that might affect scoliosis, and consent to conservative treatment. The exclusion criteria included nonidiopathic scoliosis, the presence of any contraindications to both treatments, accompanying mental or psychological problems, accompanying any chronic neurological–muscular or rheumatic diseases, accompanying other orthopedic problems, patients with incomplete general information, or patients who withdrew from the study.

Our final sample of 117 participants provided adequate statistical power, exceeding the target of 18 per group (36 total) set to account for a 20% dropout rate. This target was based on an *a priori* calculation requiring 14 participants per group (*α* = 0.05, power = 90%) to detect an MCID of 5.6° (pooled SD = 3.95°) (8).

### Interventions

#### Breaststroke combined with electroacupuncture group (BE-group)

In this group, electroacupuncture therapy was performed three times a week for three consecutive months. Meanwhile, supervised 800-m breaststroke exercise was used as a regular treatment method twice a week for at least 12 months.

Electroacupuncture was performed with Huatuo disposable acupuncture needles (0.30 × 75 mm) and a Yingdi KWD-808 Ⅱ low-frequency pulse electrotherapeutic apparatus by an acupuncturist. Patients were placed in the prone position. The main acupoints included Jiaji (EXB2) acupoints on the convex side of four vertebrae in the apical vertebral region and the bilateral Shenshu (BL23), bilateral Dachangshu (BL25), bilateral Weizhong (BL40), bilateral Xuanzhong (GB39), and bilateral Yanglingquan (GB34) acupoints. If the patient had low back pain, the Yaoyangguan (GV3) acupoint was added to the main acupoints above. If there was pain in the hip, the Zhibian (BL54) and Huantiao (GB30) acupoints on the affected side were used. If there was pain in the thigh, the Chengfu (BL36) and Fengshi (GB31) acupoints on the affected side were added. If there was pain in the lower leg, the Chengshan (BL57) and Feiyang (BL58) acupoints on the affected side were added. After sterilization, the filiform needles (GB 2024–2016) were inserted vertically to a depth of 0.5–1 cun, and then manipulated lightly by twisting them back and forth and lifting them up and down until the acupuncturist felt increased resistance to the needle (called deqi in TCM) (19). The electrodes were placed on the needle handles and stimulated for 30 min at 4 Hz with a current intensity of 4–6 mA.

#### Breaststroke group (B-group)

The patients in the B-Group were asked to exercise by supervised 800-m breaststroke twice a week for 12 months. In this group, swimming and observation were regarded as the only treatments.

To enhance adherence, all patients were scheduled for outpatient follow-up visits every 3 months. Additionally, a dedicated research assistant maintained regular contact with patients via WeChat app. Patients were instructed to send a message after completing each session of electroacupuncture or swimming. The adherence rate was calculated as the number of completed sessions divided by the number of prescribed sessions.

### Data collection

The medical records and radiographic images of all patients were analysed.

#### Demographic characteristics

Demographic data such as age, gender, weight, height, and body mass index (BMI) at baseline were collected.

#### Image evaluation

Standing anterior-posterior and lateral 36-inch radiographs of the spine were collected at three points in time: before treatment (baseline), at the 3-month follow-up, and at the 12-month follow-up. Radiographic parameters included curve type (thoracic-T, thoracolumbar-TL, lumbar-L, and S-shaped) (20), Risser sign, Cobb angle, thoracic kyphosis (TK), and lumbar lordosis (LL). To minimize the possibility of measurement errors, all radiographs were measured twice using the same protractor by one experienced orthopedic surgeon who was blinded to the patient groupings and time points. Our results are the average of these two measurements. In accordance with the SOSORT guidelines (2), we considered an increase in the Cobb angle ≥5° as “progression”, a change in the Cobb angle <5° as “stabilization”, and a reduction in the Cobb angle ≥5° as “improvement”.

#### ATR

The angle of trunk rotation was evaluated via Bunnell's scoliometer and Adam's forward bend test at three points in time. The patients were asked to bend forward, and the angle of trunk rotation (the angle between the horizontal plane and a plane across the posterior aspect of the trunk) was measured via the apical vertebrae of the main curve. To eliminate the error, the measurement was performed twice by the same examiner, and the final rotation value resulted from their mean value. This measurement has been proven to be sensitive, specific, and reliable (21).

#### SRS-22 questionnaire

The SRS-22 questionnaire was used to assess health-related quality of life (HRQoL). The questionnaires of all patients were collected at three points in time. The SRS-22 questionnaire is a valid self-reported instrument for the assessment of quality of life related to scoliosis. It includes five domains: self-image, function, pain, mental health (five questions each), and satisfaction with treatment (two questions). The questionnaire has a total of 22 items that are scored from 1 (worst) to 5 (best) for each item (22). The final score is the average of these five domains. This instrument has been found to have good validity and test-retest reliability (23).

#### Walter reed visual assessment scale (WRVAS)

The WRVAS was designed to assess the perceived physical deformity of AIS patients (24). All patients were asked to finish WRVAS at three points in time. The test allows patients to describe their perception of their deformity. The WRVAS reveals seven visible aspects of spinal deformity, including the shoulder level, body curve, head pelvis, flank prominence, rib prominence, scapular rotation, and head rib pelvis. The scores for each category range from 1 (no deformity) to 5 (the worst deformity), and the total score is generated from the sum of the scores from the seven domains. The WRVAS was found to have high reliability and validity for assessing the perception of deformities in AIS patients (25).

### Statistical analysis

All analyses were performed with SPSS (Version 26.0 software, IBM Corp., Armonk, NY, USA). Descriptive statistics are presented as the mean ± standard deviation (SD), number (*n*) or percentage (%). To compare the demographic data between the two groups, an independent samples *t* test was used for continuous variables, and a chi-square test or Fisher's exact test was used for categorical variables. For the intergroup comparisons, the continuous data, including the Cobb angle, ATR, TK, and LL, subscales of the SRS-22, and WRVAS were compared via independent samples *t* tests. To analyse the changes within the groups over time, a paired samples *t* test was applied for continuous variables. The ordinal data were analysed with the Mann‒Whitney *U* test. Statistical significance was determined at *p* < 0.05. A Bonferroni correction was applied for multiple testing. The significance level was adjusted accordingly (*α* = 0.05/number of comparisons). All statistical analyses were performed by an experienced statistician.

## Results

### Patient characteristics

**Figure 1:**
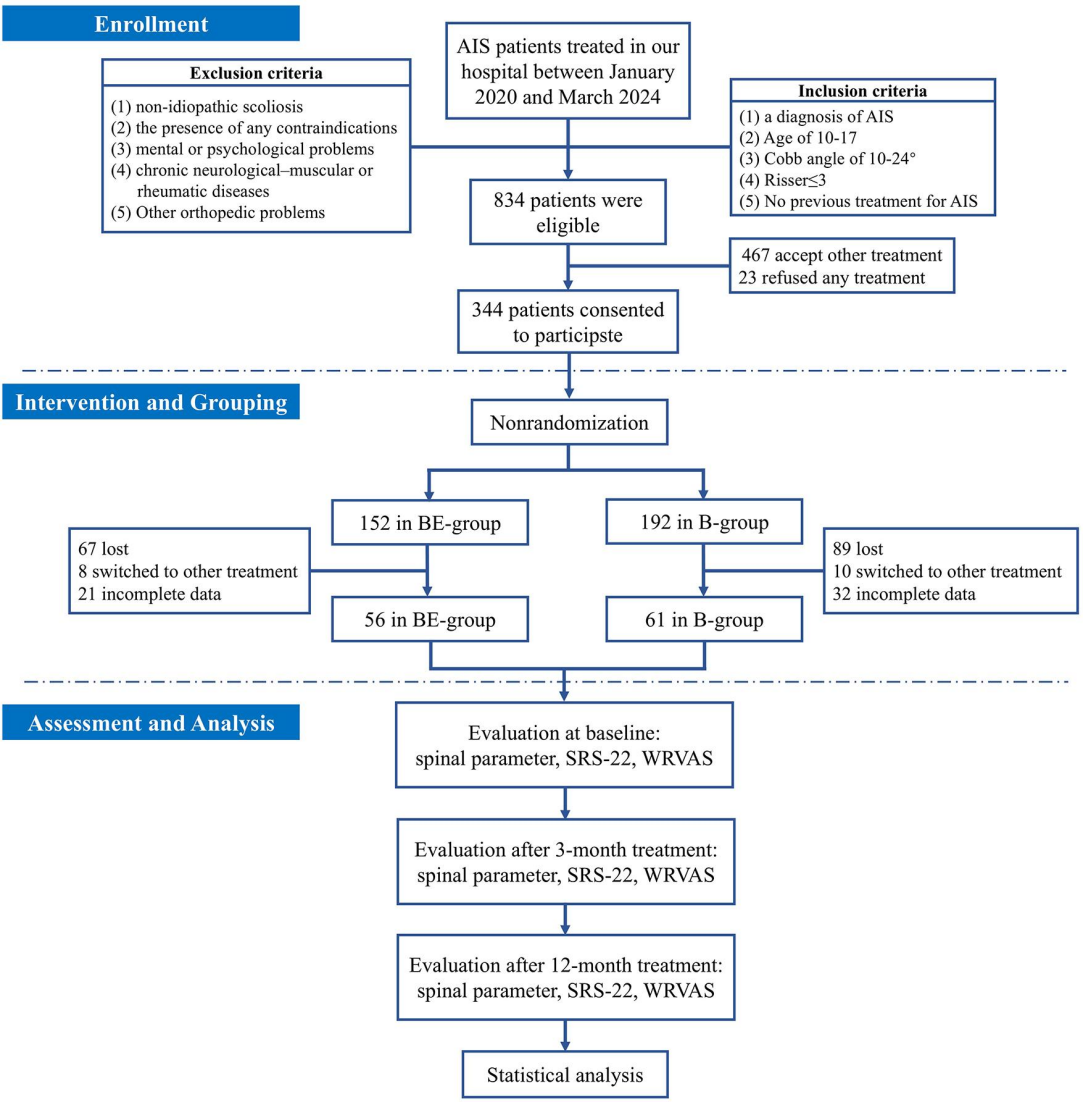
The flow diagram of this study. AIS, adolescent idiopathic scoliosis; BE, breaststroke combined with electroacupuncture therapy; B, breaststroke therapy; WRVAS, Walter reed visual assessment scale; SRS-22, Scoliosis Research Society Outcomes Questionnaire-22.

Fifty-six patients (45 females, 80.4%) in the BE-group and sixty-one (52 females, 85.2%) in the B-group were finally included in the analysis. No adverse events were reported during the one-year treatment and follow-up period. Adherence to electroacupuncture (BE-group) was 88.1 ± 8.7%. Swimming adherence was comparable between the BE-group (70.5 ± 7.3%) and the B-group (68.7 ± 7.0%) (*p* = 0.181). The study protocol details, including patient flow and dropout information, are displayed in Figure 1.

All clinical characteristics at baseline are presented in Tables 1, 2. There was no significant difference between the two groups in terms of demographic data, radiological parameters, SRS-22 scores, or WRVAS scores (*p* > 0.05). Therefore, they were homogenous and formed a good baseline for comparing the effectiveness of the two conservative treatment methods.

**Table 1:** Demographic data at baseline (mean ± SD).

Number	BE-Group	B-Group	*t*	*P* value
	*n* = 56	*n* = 61		
Age (years)	13.1 ± 1.9	12.8 ± 1.8	1.019	0.310^c^
Height (cm)	157.3 ± 12.4	159.8 ± 13.1	−1.058	0.292^c^
Weight (kg)	45.4 ± 8.3	44.7 ± 7.4	0.482	0.631^c^
BMI (kg/m^2^)	18.4 ± 2.9	17.6 ± 3.1	1.438	0.153^c^
Gender			*χ* ^2^	*P* value
Male	11 (19.6%)	9 (14.8%)	0.49	0.483^a^
Female	45 (80.4%)	52 (85.2%)		
Risser sign				*P* value
0	22 (39.3%)	28 (45.9%)		0.622^b^
Ⅰ	16 (28.6%)	15 (24.6%)		
Ⅱ	12 (21.4%)	10 (16.4%)		
Ⅲ	6 (10.7%)	8 (13.1%)		
Curve type			χ^2^	*P* value
T	21 (37.5%)	20 (32.8%)	0.28	0.594^a^
TL/L	35 (62.5%)	41(67.2%)		

BE-group, breaststroke combined with electroacupuncture group; B-group, breaststroke group; BMI, body mass index; T, thoracic curve; TL/L, thoracolumbar curve/lumbar curve; SD, standard deviation.

^a^ Chi-squared test.

^b^ Fisher's exact tests.

^c^ Independent samples *t* test.

**Table 2:** The outcomes of intergroup and intragroup comparison (mean ± SD).

Parameters	Group	Baseline	3-month	12-month	3-month vs. Baseline		12-month vs. Baseline		12-month vs. 3-month	
					P	Mean Change (95% CI)	P	Mean Change (95% CI)	P	Mean Change (95% CI)
Cobb angle	BE-group (*n* = 56)	17.5 ± 4.3	15.0 ± 4.8	14.7 ± 5.4	<0.001*^,^ **	−2.5 (−3.3/−1.7)	<0.001*^,^ **	-2.8 (−3.9/−1.7)	0.461	−0.3 (−1.0/0.5)
	B-group (*n* = 61)	17.9 ± 3.6	17.8 ± 4.1	17.6 ± 4.7	0.794	−0.1 (−0.6/0.4)	0.483	−0.3 (−1.1/0.5)	0.343	−0.2 (−0.7/0.3)
	P	0.646	0.001*^,^ **	0.003*^,^ **						
ATR	BE-group (*n* = 56)	6.9 ± 2.7	6.3 ± 2.3	6.1 ± 2.4	0.028*	−0.4 (−0.8/−0.04)	0.018*	−0.6 (−1.1/−0.1)	0.221	−0.2 (−0.4/0.1)
	B-group (*n* = 61)	7.3 ± 2.6	7.1 ± 2.6	7.3 ± 2.7	0.197	−0.1 (−0.3/0.06)	0.928	0.02 (−0.3/0.4)	0.289	0.1 (−0.1/0.4)
	P	0.312	0.084	0.019*						
TK	BE-group (*n* = 56)	20.8 ± 9.2	20.5 ± 8.0	19.7 ± 7.0	0.226	−0.3 (−0.8/0.2)	0.014*^,^ **	−1.1 (−2.0/−0.2)	0.005*^,^ **	−0.8 (−1.3/−0.3)
	B-group (*n* = 61)	22.7 ± 10.0	22.3 ± 8.5	21.8 ± 6.9	0.132	−0.3 (−0.9/0.2)	0.046*	−1.0 (−1.9/−0.02)	0.037*	−0.6 (−1.2/−0.0)
	P	0.278	0.223	0.104						
LL	BE-group (*n* = 56)	47.7 ± 7.9	48.1 ± 7.0	48.2 ± 7.3	0.377	0.3 (−0.4/1.1)	0.198	0.5 (0.3/1.2)	0.699	0.1 (−0.6/0.9)
	B-group (*n* = 61)	50.1 ± 9.3	50.3 ± 8.4	50.3 ± 7.7	0.444	0.2 (−0.3/0.8)	0.709	0.2 (−0/7/1.0)	0.849	−0.05 (−0.6/0.5)
	P	0.133	0.115	0.134						
SRS−22 (Function)	BE-group (*n* = 56)	4.57 ± 0.28	4.58 ± 0.28	4.59 ± 0.30	0.755	0.01 (−0.04/0.05)	0.312	0.03 (−0.02/0.07)	0.255	0.02 (−0.01/0.05)
	B-group (*n* = 61)	4.48 ± 0.31	4.49 ± 0.25	4.50 ± 0.27	0.795	0.01 (−0.04/0.06)	0.641	0.01 (−0.04/0.07)	0.687	0.01 (−0.03/0.04)
	P	0.117	0.081	0.065						
SRS-22 (Pain)	BE-group (*n* = 56)	4.26 ± 0.55	4.38 ± 0.45	4.44 ± 0.39	<0.001*^,^ **	0.12 (0.07/0.17)	<0.001*^,^ **	0.18 (0.09/0.28)	0.035*	0.06 (0.01/0.12)
	B-group (*n* = 61)	4.37 ± 0.52	4.42 ± 0.45	4.49 ± 0.38	0.035*	0.05 (0.01/0.10)	0.005*^,^ **	01.2 (0.04/0.20)	0.009*^,^ **	0.07 (0.02/0.12)
	P	0.271	0.596	0.497						
SRS-22 (Self-image)	BE-group (*n* = 56)	3.86 ± 0.62	4.04 ± 0.48	4.09 ± 0.51	0.002*^,^ **	0.18 (0.07/0.29)	0.003*^,^ **	0.23 (0.08/0.38)	0.095	0.05 (−0.01/0.11)
	B-group (*n* = 61)	3.89 ± 0.52	3.86 ± 0.46	3.88 ± 0.50	0.554	−0.02 (−0.10/0.05)	0.867	−0.01 (−0.13/0.11)	0.583	0.01 (−0.03/0.06)
	P	0.791	0.049*	0.027*						
SRS-22 (Mental health)	BE-group (*n* = 56)	4.40 ± 0.42	4.39 ± 0.39	4.53 ± 0.37	0.799	−0.004 (−0.03/0.02)	0.014*^,^ **	0.13 (0.03/0.24)	0.003*^,^ **	0.14 (0.05/0.22)
	B-group (*n* = 61)	4.42 ± 0.44	4.43 ± 0.38	4.50 ± 0.37	0.470*	0.01 (−0.02/0.05)	0.031*	0.09 (0.01/0.16)	0.005*^,^ **	0.07 (0.02/0.12)
	P	0.771	0.578	0.730						
SRS-22 (Satisfaction)	BE-group (*n* = 56)	–	3.55 ± 0.72	3.49 ± 0.77	–	–	–	–	0.418	−0.06 (−0.21/0.09)
	B-group (*n* = 61)	–	3.41 ± 0.60	3.47 ± 0.82	–	–	–	–	0.374	0.06 (−0.07/0.19)
	P	–	0.244	0.871						
SRS-22 (Total score)	BE-group (*n* = 56)	–	4.27 ± 0.28	4.30 ± 0.31	–	–	–	–	0.256	0.03 (−0.02/0.08)
	B-group (*n* = 61)	–	4.22 ± 0.23	4.26 ± 0.23	–	–	–	–	0.008*	0.04 (0.01/0.07)
	P	–	0.261	0.421						
WRVAS score	BE-group (*n* = 56)	14.5 ± 4.0	12.6 ± 3.3	12.4 ± 3.4	<0.001*^,^ **	−1.9 (−2.5/−1.3)	<0.001*^,^ **	−2.1 (−2.8/−1.3)	0.513	−0.2 (−0.7/0.4)
	B-group (*n* = 61)	14.9 ± 3.2	14.9 ± 3.3	15.1 ± 3.6	0.877	0.02 (−0.2/0.2)	0.388	0.2 (−0.3/0.6)	0.300	0.2 (−0.2/0.5)
	P	0.500	<0.001*^,^ **	<0.001*^,^ **						

CI, confidence interval; BE-group, breaststroke combined with electroacupuncture group; B-group, breaststroke group; ATR, angle of trunk rotation; TK, thoracic kyphosis; LL, lumbar lordosis; SRS-22, Scoliosis Research Society Outcomes Questionnaire−22; WRVAS, Walter reed visual assessment scale; SD, standard deviation.

The intergroup comparisons were tested using independent samples *t* test, and the intragroup comparisons were tested using paired samples *t* test.

* *P* < 0.05.

** *P* < 0.017 was considered significant, as we had to correct our analysis for multiple testing (*P*-value of 0.017 was calculated as: 0.05 divided by 3).

### Intergroup comparisons of radiological parameters, HRQoL, and physical deformities

The results of the intergroup comparisons of the Cobb angle, ATR, TK, LL, SRS-22, and WRVAS are shown in Table 2. The TK, LL, and SRS-22 subscales, including the function, pain, mental health, and satisfaction domains did not significantly differ between the two groups at any of the three visits. Compared with the B-group, the BE-group achieved significantly smaller Cobb angles (15.0 ± 4.8 vs. 17.8 ± 4.1, *p* = 0.001; 14.7 ± 5.4 vs. 17.6 ± 4.7, *p* = 0.003) after 3 and 12 months of treatment. A significantly lower WRVAS score (*p* < 0.001) indicated that patients in the BE-group were better satisfied with their appearance at both the 3-month and 12-month follow-ups (12.6 ± 3.3 vs. 14.9 ± 3.3, *p* < 0.001; 12.4 ± 3.4 vs. 15.1 ± 3.6, *p* < 0.001). At the 12-month follow-up, the differences between the two groups in ATR (6.1 ± 2.4 vs. 7.3 ± 2.7, *p* = 0.019) and self-image scores (4.09 ± 0.51 vs. 3.88 ± 0.50, *p* = 0.027) were not significant after adjustment for multiple comparisons, despite initial *p*-values being below 0.05.

### Intragroup comparison of radiological parameters, HRQoL, and physical deformity

The results of the intragroup comparisons are presented in Table 2. After 12 months of treatment, both groups showed significant improvements in the SRS-22 pain subscale scores (BE-group: 4.44 ± 0.39 vs. 4.26 ± 0.55, *p* < 0.001; B-group: 4.49 ± 0.38 vs. 4.37 ± 0.52, *p* < 0.001). At 12-month follow-up, the BE-group demonstrated significant improvements in the Cobb angle (14.7 ± 5.4 vs. 17.5 ± 4.3, *p* < 0.001), SRS-22 self-image (4.09 ± 0.51 vs. 3.86 ± 0.62, *p* = 0.003), SRS-22 mental health (4.53 ± 0.37 vs. 4.40 ± 0.42, *p* = 0.014), WRVAS (12.4 ± 3.4 vs. 14.5 ± 4.0, *p* < 0.001), as well as significant reduction in TK (19.7 ± 7.0 vs. 20.8 ± 9.2, *p* = 0.014). In contrast, the B-group exhibited no significant changes in these parameters. Likewise, at the 3-month follow-up, only the BE-group demonstrated improvements in the Cobb angle (*p* < 0.001), SRS-22 pain (*p* < 0.001), SRS-22 self-image (*p* = 0.002), and WRVAS scores (*p* < 0.001). In both groups, the LL and SRS-22 function subscales did not differ significantly across the three follow-ups. After 12 months of treatment, improvements in ATR (BE-group, *p* = 0.018), TK (B-group, *p* = 0.046), and SRS-22 mental health (B-group, *p* = 0.031) were not significant after multiple-testing correction, despite nominal *p*-values < 0.05.

### Effects of different curve types on treatment outcomes

Different curve types had an influence on treatment outcome, which was presented in Tables 3–5. The average Cobb angle of the patients in the BE-group decreased from 17.5° to 14.7° (*p* < 0.001). Among those patients, 55.4% (*n* = 31) had a reduction in the Cobb angle ≥5° (improvement), 42.9% (*n* = 24) had a change in the Cobb angle <5° (stabilization), and 1.8% (*n* = 1) had an increase in the Cobb angle ≥5° (progression). In the B-group (*n* = 61), the average Cobb angle decreased from 17.9° to 17.2° (*P* = 0.116). We detected that only 14.8% (*n* = 9) of patients experienced Cobb angle improvement, 80.3% (*n* = 49) experienced stabilization, and 4.9% (*n* = 3) experienced progression. There was a significant difference between the two groups (*Z* = −4.542, *p* < 0.001), which means that breaststroke combined with electroacupuncture therapy had a better effect on AIS patients than did breaststroke alone.

**Table 3:** The proportion of improvement, stabilization, and progression in All AIS at final follow-up.

Group	Improvement		Stabilization		Progression		*Z*	*P* value
	*n*	percentage	*n*	percentage	*n*	percentage		
BE-group (*n* = 56)	31	55.4%	24	42.9%	1	1.8%	−4.542	<0.001*
B-group (*n* = 61)	9	14.8%	49	80.3%	3	4.9%		

AIS, adolescent idiopathic scoliosis; BE-group, breaststroke combined with electroacupuncture group; B-group, breaststroke group.

Ordinal data were analyzed with Mann–Whitney *U* test.

* *P* < 0.05.

**Table 4:** The proportion of improvement, stabilization, and progression in T- curve AIS at final follow-up.

Group	Improvement		Stabilization		Progression		Z	*P* value
	*n*	percentage	*n*	percentage	*n*	percentage		
BE-group (*n* = 56)	7	33.3%	13	61.9%	1	4.8%	−1.591	0.112
B-group (*n* = 61)	3	15.0%	14	70.0%	3	15.0%		

AIS, adolescent idiopathic scoliosis; BE-group, breaststroke combined with electroacupuncture group; B-group, breaststroke group; T-curve, thoracic curve.

Ordinal data were analyzed with Mann–Whitney *U* test.

**Table 5:** The proportion of improvement, stabilization, and progression in TL/L-curve AIS at final follow-up.

Group	Improvement		Stabilization		Progression		*Z*	*P* value
	*n*	percentage	*n*	percentage	*n*	percentage		
BE-group (*n* = 56)	24	68.6%	11	31.4%	0	0%	−4.763	<0.001*
B-group (*n* = 61)	6	14.6%	35	85.4%	0	0%		

AIS, adolescent idiopathic scoliosis; BE-group, breaststroke combined with electroacupuncture group; B-group, breaststroke group; TL/L-curve, thoracolumbar/lumbar curve.

Ordinal data were analyzed with Mann–Whitney *U* test.

* *P* < 0.05.

To further evaluate the effect of curve type on treatment outcomes, we divided AIS patients into subgroups according to the curve type. For the T-curve (thoracic curve) AIS (Table 4), among the 21 patients in the BE-group, 33.3% (*n* = 7) improved by ≥5°, 61.9% (*n* = 13) stabilized, and 4.8% (*n* = 1) progressed ≥5°. Among those in the B-group (*n* = 20), 15.0% (*n* = 3) experienced improvement, 70.0% (*n* = 14) experienced stabilization, and 15.0% (*n* = 3) experienced progression. For the T-curve AIS, there were no significant differences in treatment outcomes between the two groups (Z = −1.591, *P* = 0.112).

Table 5 shows the results for AIS patients with a TL-curve (thoracolumbar curve)/L-curve (lumbar curve). In the BE-group (*n* = 35), 68.6% (*n* = 24) of patients experienced Cobb angle improvement, 31.4% (*n* = 11) experienced stabilization, and no patients experienced progression. Among those in the B-group (*n* = 41), 14.6% (*n* = 6) experienced improvement, 85.4% (*n* = 35) experienced stabilization, and no patients experienced progression. AIS patients with TL/L-curve exhibited significant intergroup differences in treatment outcomes (Z = −4.763, *P* < 0.001). These subgroup analyses, which were not pre-specified and have limited sample sizes, should be interpreted with caution. However, given the extremely small *p*-value (*p* < 0.001), the conclusion still holds a certain reference value.

## Discussion

### Summary

The results of this study demonstrated that breaststroke combined with electroacupuncture therapy may be safe and effective in correcting deformities and improving the quality of life of mild AIS patients.

### Clinical context

In 2018, SOSORT proposed that the basic goals of conservative treatment for AIS are to stop curve progression at puberty, prevent respiratory dysfunction, treat spinal pain, and improve aesthetics (2). For mild AIS, orthopaedists may recommend observation with regular outpatient follow-up as the only treatment. However, scoliosis may progress rapidly in a very short time. Therefore, early intervention is important for AIS patients.

### Swimming role

It has been reported that nonagonistic sports activities have a protective effect against scoliosis progression in adolescents (13). The SOSORT recommends that patients with scoliosis remain active in sports activities (2). However, the effectiveness of exercise has not been fully accepted by orthopedists or society. A critical limitation is the paucity of high-quality studies investigating which sport is effective against curve progression. In our study, we suggested 800-m-long breaststroke twice a week as the key sport intervention for mild AIS patients. After the 12-month follow-up, both the Cobb angle and the ATR remained stable without significant progression. Up to 80.3% of the patients in the swimming group maintained curve stability, 14.8% improved, and only 4.9% progressed. When compared to the established natural history of mild AIS—which shows a progression rate of up to 26% within one year in untreated, skeletally immature patients (26)—our results suggest that breaststroke may help prevent curve progression but has limited efficacy in correcting deformities. Another study, which followed patients until skeletal maturity or surgery, reported a curve progression rate as high as 65% (27). Given this high rate of progression, the authors suggested that early intervention is warranted in skeletally immature individuals with mild AIS.

In fact, the impact of swimming on scoliosis is controversial. Some studies have shown that competitive swimming is associated with a greater risk of developing scoliosis in adolescents (28, 29). On the other hand, some scholars hold the opposite opinion. Barczyk et al. reported that swimming and corrective exercise in water could reduce the Cobb angle, TK, and LL (30). Bielec et al. reported that regular participation in swimming classes once a week had significant corrective effects on scoliosis (31). Gonen et al. reported that swimming maintained scoliosis stability (with no progression or improvement) in adolescent swimmers at the 1-year follow-up (32). Similarly, in the breaststroke group of our study, the majority of patients also demonstrated comparable stability without progression after one year of intervention. In fact, irrespective of the controversial effect on curve progression, swimming also has a positive effect on pulmonary function, muscle strength, mental health, and even bone development (16, 17, 33). These advantages of swimming may help achieve the goals of conservative treatment for AIS. Additionally, swimming has also been suggested as a recommended treatment for chronic nonspecific low back pain (34). By reviewing the literature, Ribaud et al. reported that moderate but regular swimming could maintain fitness and control pain in chronic low back pain patients (15). Breaststroke represents a symmetrical exercise modality and promotes balanced development of the bilateral muscles. In our study, breaststroke was exclusively selected as the therapeutic swimming style because of its biomechanical advantages. Consistent with prior findings, 12 months of breaststroke training in the B-group yielded significant improvements in the SRS-22 pain domain. Despite losing statistical significance after multiple-testing correction, the observed improvement in SRS-22 mental health and reduction in TK suggest a potential beneficial role for swimming in these domains. Although swimming has been considered a treatment option for scoliosis, a cross-sectional study of adolescent competitive swimmers revealed a higher prevalence of LBP in females and an increased risk of trunk asymmetry and hyperkyphosis (29). We speculate that the difference in treatment outcomes may be related to the intensity and form of swimming.

### Electroacupuncture role

Acupuncture has been widely used to treat diseases and relieve pain in China for three thousand years. Recent reports suggest that acupuncture can be helpful in the treatment of scoliosis (35). TCM suggests that acupuncture needle stimulation techniques can tonify and sedate energy in meridians. It is believed that tonifying the areas of weak muscles and sedating the areas of overactive muscles can have relaxing and balancing effects (7). Weiss et al. conducted a controlled single-blind crossover study and demonstrated that acupuncture could improve the surface rotation of AIS patients by no more than 35 degrees (36). Liu et al. reported that acupuncture not only plays an important role in pain control but also reduces the Cobb angle in patients with degenerative scoliosis (37). A systematic review including 489 patients with scoliosis from six studies concluded that the addition of acupuncture to other forms of routine care could be effective against mild scoliosis, especially in reducing the Cobb angle (38). Acupuncture has been proven to be safe and a good conservative treatment option (39).

Electroacupuncture therapy is a combination of electrical stimulation, acupuncture, and the theory of meridians and acupoints. In many aspects, electroacupuncture is more effective than manual acupuncture (40, 41). Lu et al. demonstrated that electroacupuncture could effectively improve scoliosis secondary to Parkinson's disease with few side effects and a low risk of recurrence (42). In China, electroacupuncture is often used to treat scoliosis and has proven to be an effective intervention, but the quality of evidence is low. In addition, acupuncture combination treatment tends to improve symptoms of scoliosis better than acupuncture monotherapy does (38). In our study, we proposed a novel therapeutic strategy including electroacupuncture and breaststroke for mild AIS. During the 1-year treatment period, the combined therapy group received 3 months of electroacupuncture and 12 months of supervised breaststroke training, whereas the breaststroke group received only 12 months of breaststroke training. After treatment, the intragroup comparison revealed significant improvements in the Cobb angle in the combined therapy group. Compared with those of the B-group, the Cobb angle of the BE-group were significantly smaller, which indicates that electroacupuncture plays a protective role in treating scoliosis. It should be noted that due to the conservative nature of the Bonferroni correction, the between-group and within-group comparisons for ATR did not retain their statistical significance after adjustment, despite initial *p*-values being below 0.05. However, the Cobb angle, which is the primary outcome measure for AIS, remained statistically significant even after this rigorous adjustment. Our results revealed that more than half (55.4%) of the patients in the BE-group experienced curve improvement, 42.9% remained stable, and only 1.8% experienced progression at the 12-month evaluation. This result was better than that in the B-group. The comparison also suggested that breaststroke combined with electroacupuncture could not only prevent curve progression but also effectively reduce the degree of deformity. In the BE-group, the SRS-22 self-image subscale and the WRVAS at both the 3-month and 12-month evaluations were significantly improved, and the results were consistent with the changes in the scoliosis parameters. Interestingly, although no significant intergroup differences were observed in the pain or mental health subscale scores, both indicators showed improvement compared to baseline within each group. Despite not surviving multiple-testing correction, the mental health improvement in the B-group (*p* = 0.031) hints at a positive role for swimming. Some reasons could explain this result. Physical activity could play a beneficial role in the mental health of adolescents (43). In our study, patients in both groups swam twice a week, and swimming was also effective in generating positive changes in mental health (44). Many studies have confirmed that electroacupuncture is an effective conservative treatment to relieve pain via different meridians and acupoints according to symptoms (45–47). As mentioned above, swimming is also an effective method for reducing pain (15, 34, 48). Given these results, it is more reasonable to recommend breaststroke combined with electroacupuncture to treat mild AIS.

### Curve types

To further explore the optimal target population of this new treatment, we divided AIS patients into two subgroups: the thoracic curve (T-curve) group and the thoracolumbar/lumbar curve (TL/L-curve) group. Strube et al. demonstrated that curve location influences the outcome of conservative treatment for AIS. Biomechanical studies have shown that the flexibility of the lumbar spine is superior to that of the thoracic spine (49). The lumbar spine, which is mainly stabilized by muscles, is flexible, whereas the thoracic spine surrounded by the rib cage is rigid and stable (50–52). In our study, no significant difference was detected between the BE-group and the B-group among the AIS patients with T-curve. In contrast, within the TL/L-curve group, the combined treatment was far more effective than swimming alone, with up to 68.6% of patients improving, and no patients worsened. This result demonstrated that breaststroke combined with electroacupuncture was more suitable for AIS patients with TL/L curves. These findings may be associated with the theory that the lumbar spine is mainly stabilized by core muscles. Both electroacupuncture and swimming work mainly by affecting muscles. Acupuncture can increase blood flow, cause microenvironmental changes, and strengthen muscles (53–55). Breaststroke represents a symmetrical exercise modality that can train the core muscle and promote balanced development of the bilateral muscle. Acupuncture-induced changes in the muscular microenvironment may further enhance the therapeutic effects of breaststroke.

### Limitations and strengths

The results of our study should be considered in the context of several limitations. First, its retrospective design may introduce selection bias. Although baseline characteristics were similar between groups, non-randomized treatment allocation could confound the outcomes. Second, the lack of blinding for participants and clinicians may have affected subjective patient-reported outcomes, possibly amplifying perceived benefits in the combined therapy group. To minimize bias in image assessments, radiographic parameters were measured by an assessor blinded to group allocation. Lastly, despite controlling for known confounders, the impact of unmeasured factors cannot be excluded.

This study's limitations are offset by key strengths, notably the demonstration that electroacupuncture significantly enhances the corrective effect of breaststroke in AIS. This finding not only validates the combined regimen but also justifies further exploration of traditional Chinese medicine approaches, such as acupuncture and chiropractic care, for managing this condition. This is a preliminary study, and future prospective, randomized, and blinded trials are needed to confirm our results.

## Conclusions

The present study introduced a novel conservative treatment strategy for skeletally immature patients with mild AIS: breaststroke combined with electroacupuncture therapy. Our study found that whereas isolated breaststroke effectively maintained curve stability, combining it with electroacupuncture provided superior corrective efficacy. In addition, this combined approach may be particularly beneficial for AIS patients with TL/L curves.

## Data Availability

The original contributions presented in the study are included in the article/Supplementary Material, further inquiries can be directed to the corresponding authors.

## References

[1] Hresko MT. Clinical practice. Idiopathic scoliosis in adolescents. N Engl J Med. (2013) 368(9):834–41. 10.1056/NEJMcp1209063 23445094

[2] Negrini S Donzelli S Aulisa AG Czaprowski D Schreiber S de Mauroy JC 2016 Sosort guidelines: orthopaedic and rehabilitation treatment of idiopathic scoliosis during growth. Scoliosis Spinal Disord. (2018) 13(1):3. 10.1186/s13013-017-0145-8 29435499 PMC5795289

[3] Richards BS Bernstein RM D’Amato CR Thompson GH. Standardization of criteria for adolescent idiopathic scoliosis brace studies: srs committee on bracing and nonoperative management. Spine (Phila Pa 1976) (2005) 30(18):2068–75, 2076–77. 10.1097/01.brs.0000178819.90239.d0 16166897

[4] Ng P Claus A Izatt MT Pivonka P Tucker K. Is spinal neuromuscular function asymmetrical in adolescents with idiopathic scoliosis compared to those without scoliosis?: A narrative review of surface EMG studies. J Electromyogr Kinesiol. (2022) 63:102640. 10.1016/j.jelekin.2022.102640 35219074

[5] Jiang J Meng Y Jin X Zhang C Zhao J Wang C Volumetric and fatty infiltration imbalance of deep paravertebral muscles in adolescent idiopathic scoliosis. Med Sci Monit. (2017) 23:2089–95. 10.12659/msm.902455 28461686 PMC5424650

[6] Lu L Zhang Y Tang X Ge S Wen H Zeng J Evidence on acupuncture therapies is underused in clinical practice and health policy. Br Med J. (2022) 376:e067475. 10.1136/bmj-2021-067475 35217525 PMC8868048

[7] Boehland T Montgomery AD Mortenson M. Combination acupuncture and cupping for treating adult idiopathic scoliosis. Med Acupunct. (2020) 32(4):229–33. 10.1089/acu.2020.1411 32879649 PMC7455474

[8] Jia K Yang Y. Effect of acupuncture and massage on adolescent idiopathic scoliosis and pain severity. Front Med (Lausanne). (2025) 12:1613800. 10.3389/fmed.2025.1613800 40761866 PMC12319020

[9] Chu EC Lee WT Tam D Ng NY Nur AB. Scoliosis causing cervical dystonia in a chiropractic office. Cureus. (2023) 15(3):e35802. 10.7759/cureus.35802 36891176 PMC9986506

[10] Pu CE Chakkaravarthy DM Huang K Ho V Lo FS Bhaumik A. Changes in radiographic parameters following chiropractic treatment in 10 patients with adolescent idiopathic scoliosis: a retrospective chart review. Clin Pract. (2020) 10(3):1258. 10.4081/cp.2020.1258 32952984 PMC7482186

[11] Zhang B Shi H Cao S Xie L Ren P Wang J Revealing the magic of acupuncture based on biological mechanisms: a literature review. Biosci Trends. (2022) 16(1):73–90. 10.5582/bst.2022.01039 35153276

[12] Langevin HM Schnyer R MacPherson H Davis R Harris RE Napadow V Manual and electrical needle stimulation in acupuncture research: pitfalls and challenges of heterogeneity. J Altern Complement Med. (2015) 21(3):113–28. 10.1089/acm.2014.0186 25710206 PMC4855731

[13] Negrini A Donzelli S Vanossi M Poggio M Cordani C Zaina F Sports participation reduces the progression of idiopathic scoliosis and the need for bracing. An observational study of 511 adolescents with risser 0–2 maturation stage. Eur J Phys Rehabil Med. (2023) 59(2):222–27. 10.23736/S1973-9087.23.07489-0 36892518 PMC10167700

[14] Barczyk K Skolimowski T Zawadzka D. Changes in body posture in children with first-degree scoliosis taking part in corrective exercises in a water environment. Ortop Traumatol Rehabil. (2005) 7(2):180–86. 17615512

[15] Ribaud A Tavares I Viollet E Julia M Herisson C Dupeyron A. Which physical activities and sports can be recommended to chronic low back pain patients after rehabilitation? Ann Phys Rehabil Med. (2013) 56(7–8):576–94. 10.1016/j.rehab.2013.08.007 24140440

[16] Silvestri M Crimi E Oliva S Senarega D Tosca MA Rossi GA Pulmonary function and airway responsiveness in young competitive swimmers. Pediatr Pulmonol. (2013) 48(1):74–80. 10.1002/ppul.22542 22431206

[17] Tang Z Wang Y Liu J Liu Y. Effects of aquatic exercise on mood and anxiety symptoms: a systematic review and meta-analysis. Front Psychiatry. (2022) 13:1051551. 10.3389/fpsyt.2022.1051551 36465296 PMC9714032

[18] Heshmati S Ghahraman Tabrizi K Daneshjoo A Hosseini E Bahiraei S Sahebozamani M Effects of asymmetric and symmetric sport load on upper and lower extremity strength and balance: a comparison between the dominant and non-dominant side in adolescent female athletes. Sports (Basel). (2025) 13(3):89. 10.3390/sports13030089 40137813 PMC11945827

[19] Yang XY Shi GX Li QQ Zhang ZH Xu Q Liu CZ. Characterization of deqi sensation and acupuncture effect. Evid Based Complement Alternat Med (2013) 2013:319734. 10.1155/2013/319734 23864884 PMC3705793

[20] Friedman B Ponseti IV. Prognosis in idiopathic scoliosis. J Bone Joint Surg Am. (1950) 32A(2):381–95. 10.2106/00004623-195032020-00017 15412180

[21] Amendt LE Ause-Ellias KL Eybers JL Wadsworth CT Nielsen DH Weinstein SL. Validity and reliability testing of the scoliometer. Phys Ther. (1990) 70(2):108–17. 10.1093/ptj/70.2.108 2296610

[22] Cheung KM Senkoylu A Alanay A Genc Y Lau S Luk KD. Reliability and concurrent validity of the adapted Chinese version of scoliosis research society-22 (SRS-22) questionnaire. Spine (Phila Pa 1976). (2007) 32(10):1141–45. 10.1097/01.brs.0000261562.48888.e3 17471100

[23] Asher M Min LS Burton D Manna B. The reliability and concurrent validity of the scoliosis research society-22 patient questionnaire for idiopathic scoliosis. Spine (Phila Pa 1976). (2003) 28(1):63–9. 10.1097/00007632-200301010-00015 12544958

[24] Sanders J Polly DW Cats-Baril W Jones J Lenke LG O’brien MF Analysis of patient and parent assessment of deformity in idiopathic scoliosis using the Walter reed visual assessment scale. Spine (Philadelphia, Pa. 1976). (2003) 28(18):2158–63. 10.1097/01.BRS.0000084629.97042.0B 14501929

[25] Pineda S Bago J Gilperez C Climent JM. Validity of the Walter reed visual assessment scale to measure subjective perception of spine deformity in patients with idiopathic scoliosis. Scoliosis. (2006) 1(1):18. 10.1186/1748-7161-1-18 17090338 PMC1654183

[26] Modi HN Suh S Yang J Hong J Venkatesh KP Muzaffar N. Spontaneous regression of curve in immature idiopathic scoliosis—does spinal column play a role to balance? An observation with literature review. J Orthop Surg Res. (2010) 5(1):80. 10.1186/1749-799X-5-80 21047435 PMC2992045

[27] Zapata KA Sucato DJ Lee MC Jo C. Skeletally immature patients with adolescent idiopathic scoliosis curves 15°–24° are at high risk for progression. Spine Deform. (2019) 7(6):870–74. 10.1016/j.jspd.2019.02.012 31731996

[28] Ghanem I Rizkallah M. Adolescent idiopathic scoliosis for the primary care physician: frequently asked questions. Curr Opin Pediatr. (2019) 31(1):48–53. 10.1097/MOP.0000000000000705 30461512

[29] Zaina F Donzelli S Lusini M Minnella S Negrini S. Swimming and spinal deformities: a cross-sectional study. J Pediatr. (2015) 166(1):163–67. 10.1016/j.jpeds.2014.09.024 25444007

[30] Barczyk K Zawadzka D Hawrylak A Bochenska A Skolimowska B Malachowska-Sobieska M. The influence of corrective exercises in a water environment on the shape of the antero-posterior curves of the spine and on the functional status of the locomotor system in children with IO scoliosis. Ortop Traumatol Rehabil. (2009) 11(3):209–21. 19777685

[31] Bielec G Peczak-Graczyk A Waade B. Do swimming exercises induce anthropometric changes in adolescents? Issues Compr Pediatr Nurs. (2013) 36(1–2):37–47. 10.3109/01460862.2013.777818 23597276

[32] Aydin C G Oner A Hekim HH Arslan AS Oztas D Akman YE The prevalence of scoliosis in adolescent swimmers and the effect of swimming on adolescent idiopathic scoliosis. Spor Hekimliği Dergisi. (2020) 55(3):200–06. 10.5152/tjsm.2020.176

[33] Albuquerque RBD Geraldes AAR Rangoussis B Fonseca FDS Nascimento Neto DDC Oliveira ACCD. Swimming and bone mineral density: a sport without osteogenic stimulation? Rev Bras Med Esporte. (2020) 26(2):113–16. 10.1590/1517-869220202602216728

[34] Dwivedy S Swami VNIO. Effect of swimming as a hydrotherapeutic intervention for the management of chronic non-specific low back pain—an experimental study. Int J Adv Res (Indore). (2018) 6(8):665–75. 10.21474/IJAR01/7566

[35] Choi S Jo H Park S Sung W Keum D Kim E. The effectiveness and safety of acupuncture for scoliosis: a protocol for systematic review and/or meta-analysis. Medicine (Baltimore). (2020) 99(50):e23238. 10.1097/MD.0000000000023238 33327244 PMC7738006

[36] Weiss H Bohr S Jahnke A Pleines S. Acupucture in the treatment of scoliosis—a single blind controlled pilot study. Scoliosis. (2008) 3(1):4. 10.1186/1748-7161-3-4 18226193 PMC2266704

[37] Liu C Chen K Chiu EHH. Adult degenerative scoliosis treated by acupuncture. J Altern Complement Med (New York, N.Y.). (2009) 15(8):935–37. 10.1089/acm.2008.0515 19678786

[38] Choi S Jo H Moon J Jang J Park S Sung W Effectiveness of acupuncture for scoliosis: a systematic review. J Acupunct Res. (2022) 39(1):17–28. 10.13045/jar.2021.00325

[39] White A. The safety of acupuncture techniques. J Altern Complement Med. (2007) 13(1):9–10. 10.1089/acm.2006.6414 17309368

[40] Ulett GA Han S Han JS. Electroacupuncture: mechanisms and clinical application. Biol Psychiatry. (1998) 44(2):129–38. 10.1016/s0006-3223(97)00394-6 9646895

[41] Han QQ Fu Y Le JM Ma YJ Wei XD Ji HL The therapeutic effects of acupuncture and electroacupuncture on cancer-related symptoms and side-effects. J Cancer. (2021) 12(23):7003–09. 10.7150/jca.55803 34729102 PMC8558649

[42] Lu WJ Fan JQ Yan MY Mukaeda K Zhuang LX Wang LL. Effect of electroacupuncture for Pisa syndrome in Parkinson’s disease: a case report. World J Clin Cases. (2022) 10(30):11023–30. 10.12998/wjcc.v10.i30.11023 36338234 PMC9631139

[43] Molcho M Gavin A Goodwin D. Levels of physical activity and mental health in adolescents in Ireland. Int J Environ Res Public Health. (2021) 18(4):1713. 10.3390/ijerph18041713 33578906 PMC7916674

[44] Jackson M Kang M Furness J Kemp-Smith K. Aquatic exercise and mental health: a scoping review. Complement Ther Med. (2022) 66:102820. 10.1016/j.ctim.2022.102820 35218906

[45] Heo I Shin BC Cho JH Ha IH Hwang EH Lee JH Multicentre randomised controlled clinical trial of electroacupuncture with usual care for patients with non-acute pain after back surgery. Br J Anaesth. (2021) 126(3):692–99. 10.1016/j.bja.2020.10.038 33341226

[46] Mao JJ Liou KT Baser RE Bao T Panageas KS Romero S Effectiveness of electroacupuncture or auricular acupuncture vs usual care for chronic musculoskeletal pain among cancer survivors: the peace randomized clinical trial. JAMA Oncol. (2021) 7(5):720–27. 10.1001/jamaoncol.2021.0310 33734288 PMC7974834

[47] Onuora S. Intensive electroacupuncture reduces oa pain. Nat Rev Rheumatol. (2021) 17(1):2. 10.1038/s41584-020-00556-0 33268838

[48] Fernandes G Jennings F Nery CM Pirozzi BA Natour J. Swimming improves pain and functional capacity of patients with fibromyalgia: a randomized controlled trial. Arch Phys Med Rehabil. (2016) 97(8):1269–75. 10.1016/j.apmr.2016.01.026 26903145

[49] Strube P Gunold M Muller T Leimert M Sachse A Pumberger M Influence of curve morphology and location on the efficacy of rigid conservative treatment in patients with adolescent idiopathic scoliosis. Bone Joint J. (2021) 103-B(2):373–81. 10.1302/0301-620X.103B2.BJJ-2020-1113.R2 33517722

[50] Hajihosseinali M Arjmand N Shirazi-Adl A Farahmand F Ghiasi MS. A novel stability and kinematics-driven trunk biomechanical model to estimate muscle and spinal forces. Med Eng Phys. (2014) 36(10):1296–304. 10.1016/j.medengphy.2014.07.009 25074649

[51] Oxland TR. Fundamental biomechanics of the spine–what we have learned in the past 25 years and future directions. J Biomech. (2016) 49(6):817–32. 10.1016/j.jbiomech.2015.10.035 26706717

[52] Panjabi MM White AR. Basic biomechanics of the spine. Neurosurgery. (1980) 7(1):76–93. 10.1227/00006123-198007000-00014 7413053

[53] Takayama S Watanabe M Kusuyama H Nagase S Seki T Nakazawa T Evaluation of the effects of acupuncture on blood flow in humans with ultrasound color Doppler imaging. Evid Based Complement Alternat Med (2012) 2012:513638. 10.1155/2012/513638 22778772 PMC3388479

[54] Kim SY Min S Lee H Cheon S Zhang X Park JY Changes of local blood flow in response to acupuncture stimulation: a systematic review. Evid Based Complement Alternat Med. (2016) 2016(1):9874207. 10.1155/2016/9874207 27403201 PMC4923553

[55] Liu SY Hsieh CL Wei TS Liu PT Chang YJ Li TC. Acupuncture stimulation improves balance function in stroke patients: a single-blinded controlled, randomized study. Am J Chin Med. (2009) 37(3):483–94. 10.1142/S0192415X09006990 19606509

